# Unilateral Distribution of Lichen Planus Pigmentosus-Inversus Unresponsive to Clobetasol and Hydroquinone

**DOI:** 10.7759/cureus.17368

**Published:** 2021-08-22

**Authors:** Vanessa L Ramos, Lauren R Mason, Scott M Whitlock, Robert J Pariser

**Affiliations:** 1 Dermatology, Eastern Virginia Medical School, Norfolk, USA

**Keywords:** lichen planus pigmentosus-inversus, clobetasol, hydroquinone, lichen planus, hyperpigmentation

## Abstract

Lichen planus pigmentosus-inversus (LPP-inversus) is a rare, pigmented variant of lichen planus of unknown etiology. This skin condition typically affects the intertriginous and flexural regions of the body bilaterally. We report an unusual case presentation with unilateral distribution of LPP-inversus in a woman originally from Nepal. The lesions developed rapidly over a three-month period and were recalcitrant to therapy with topical clobetasol and hydroquinone.

## Introduction

Lichen planus pigmentosus (LPP) is a rare, pigmented variant of lichen planus, characterized by symmetric, gray to dark brown macules or patches primarily affecting sun-exposed areas such as the face and neck. LPP-inversus is a term used for LPP lesions located on sun-hidden areas such as intertriginous and flexural regions [1]. Pock et al. first described LPP-inversus in 2001 [2], and a few cases have been reported subsequently. Currently, there is no consistently effective treatment for this skin condition. Here, we report an atypical case of LPP-inversus showing unilateral distribution in a Nepalese woman. The skin changes were resistant to treatment with topical corticosteroids and hydroquinone. This case is presented for educational purposes and to add to the literature regarding the management of this entity.

## Case presentation

**Figure 1:**
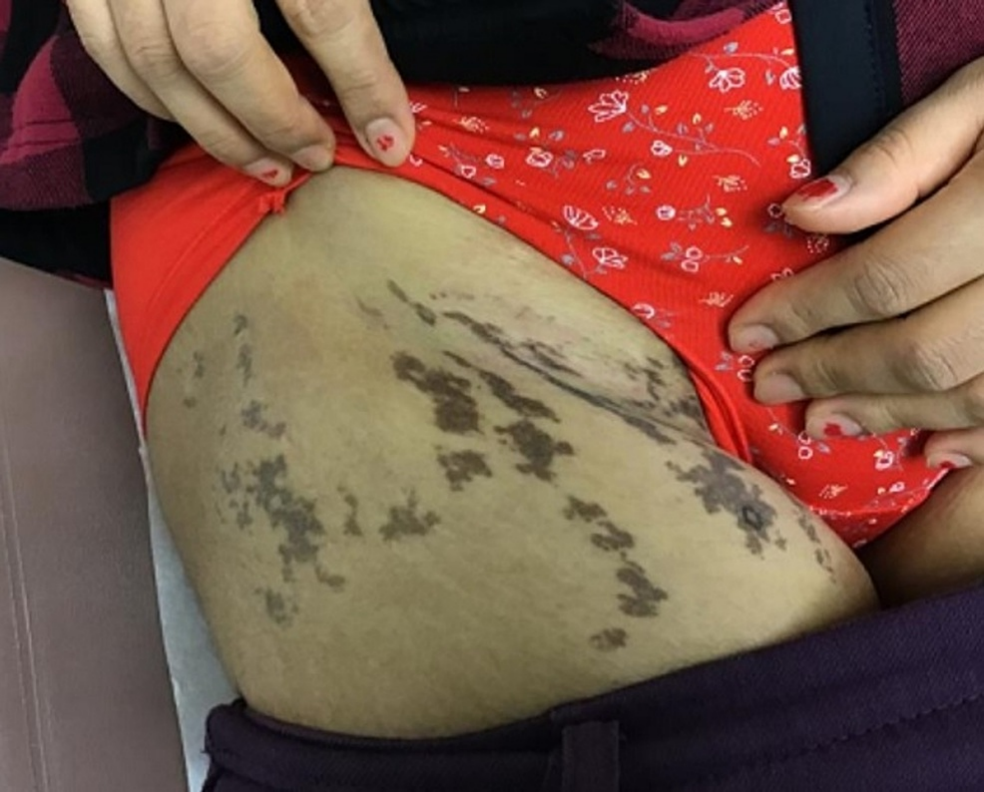
Multiple well-demarcated hyperpigmented macules and patches on the right proximal thigh and inguinal fold.

**Figure 2:**
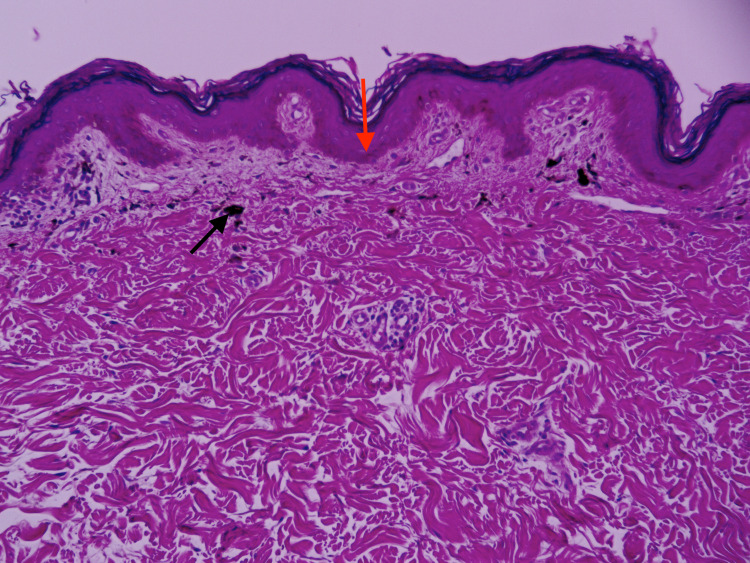
Mild atrophy of the epidermis (red arrow) with basal vacuolar change; lichenoid, lymphocytic infiltrate, prominent pigment incontinence (black arrow), and rare Civatte bodies within the papillary dermis (H&E, x100).

**Figure 3:**
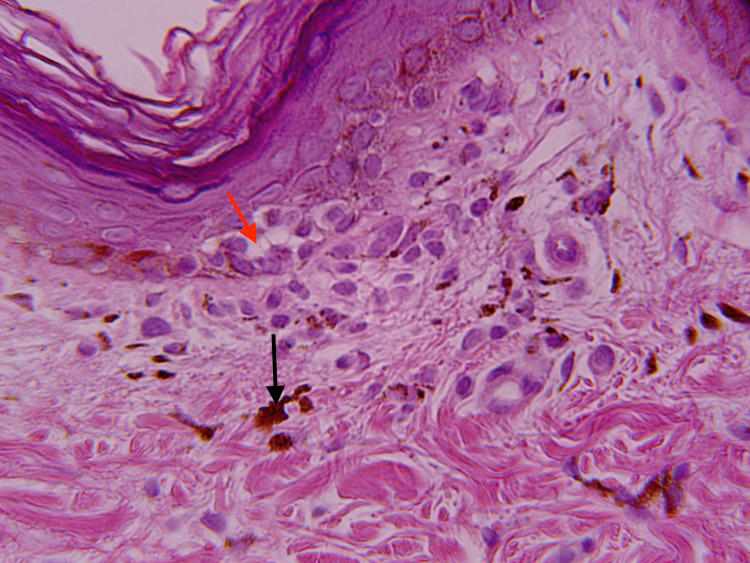
Higher magnification shows vacuolar changes in the basal layer (red arrow) and lichenoid, lymphocytic infiltrate with prominent pigment incontinence (black arrow) in the superficial dermis (H&E, x400).

A 32-year-old Nepalese woman with a history of hypercholesterolemia presented with a dark-brown, pruritic rash on her right upper thigh and groin. The rash had rapidly developed over three months without any known triggers. The patient denied any new exposures or medications and had no known allergies. Physical examination revealed multiple well-demarcated, hyperpigmented macules and patches on the right proximal thigh and inguinal fold. The lesions had a “splatter-like” arrangement along Blaschko lines (Figure 1). Nail, scalp, or oral lesions were absent. On initial clinical impression, the lesions were suspicious for post-inflammatory hyperpigmentation. The patient underwent a 4 mm punch biopsy. Histopathology showed a lichenoid, lymphocytic infiltrate with prominent pigment incontinence and rare Civatte bodies within the superficial dermis and slight atrophy of the epidermis with basal vacuolar changes (Figure 2 and Figure 3). These findings, along with the distribution of lesions, were consistent with a diagnosis of LPP-inversus. The patient was initially prescribed clobetasol propionate 0.5% cream with minimal improvement. She was subsequently initiated on hydroquinone 4% external cream, which she used for three months with no success.

## Discussion

LPP-inversus is less common and clinically distinct from classic LPP as it predominantly occurs in non-sun-exposed, intertriginous, and flexural areas such as the axilla and groin [1]. In 2001, Pock et al. initially proposed the designation of this entity after discovering seven patients with primarily flexural involvement in the Czech Republic [2]. In most cases, LPP-inversus affects areas bilaterally, appearing as asymptomatic to mildly pruritic, violaceous‐gray to hyperpigmented, dark brown round or linear macules or patches [1]. Unilateral presentation like our patient is infrequent, although few cases have been reported in the literature [3-5].

As LPP-inversus is a relatively rare disease, the exact prevalence is still unknown. Based upon limited data, this skin condition has variable epidemiology with an age of presentation ranging from 5 to 84 years [6,7]. Pediatric cases are rare, and the majority of LPP-inversus cases occur in individuals over 40 years of age [1,8]. To date, there have been mixed reports on sex predominance, but a recent review concludes that there is a female predominance [1]. Classic LPP has a higher incidence in darker-skinned individuals of Indian and Middle Eastern descent. In contrast, most cases of LPP-inversus have been reported in people with lighter skin, such as Caucasians and Asians [9]. Our patient, originally from Nepal, has moderately pigmented skin (Fitzpatrick type IV).

The etiology of LPP-inversus is not completely understood. The proposed pathophysiology involves direct T lymphocyte-mediated cytotoxic destruction of basal keratinocytes leading to inflammatory changes and pigment incontinence [5,10]. Chronic friction and tight-fitting clothing (Koebner phenomenon) have been suggested to be triggering factors. Although no causal relationship has been identified, manifestations of LPP-inversus have been reported in association with hepatitis C, lichen planopilaris, and anti-hypertensive medications [5,7].

Clinical differential diagnosis includes erythrasma, acanthosis nigricans, ashy dermatosis, post-inflammatory hyperpigmentation, and fixed drug eruption. A biopsy is helpful in confirming the diagnosis of LPP-inversus. Histopathologically, LPP-inversus appears similar to lichen planus with band-like lymphohistiocytic infiltrate in the papillary dermis with interface vacuolar changes; however, LPP-inversus may show a regressive pattern with epidermal atrophy and minimal-to-absent hyperkeratosis and hypergranulosis. In addition, pigment incontinence is prominent [1].

LPP-inversus has a benign but recalcitrant clinical course; occasionally, lesions resolve spontaneously. There are currently no standardized guidelines for the management of this condition. Treatments such as topical corticosteroids, oral corticosteroids, and topical tacrolimus have produced inconsistent results [1]. Dimova et al. used narrow-band ultraviolet B phototherapy twice weekly, with complete resolution after 20 sessions [11]. Chen et al. cited literature supporting the efficacy of combination therapy with Chinese herbs and acitretin after three cases of LPP-inversus showed progressive improvement without adverse effects [5]. Hydroquinone cream has been successfully used as a depigmenting agent in classic LPP [1]. To our knowledge, this is the first reported instance of hydroquinone being used specifically for a patient with LPP-inversus.

## Conclusions

We describe a case of unilateral presentation of LPP-inversus resistant to topical clobetasol and hydroquinone in a Nepalese woman. Our results suggest that these two treatments may not be effective for every patient with this skin condition. Additional studies on the etiology and pathophysiology of LPP-inversus may lead to more targeted treatments and improved patient outcomes.
